# Artificial Intelligence Applications in Dermatology: Where Do We Stand?

**DOI:** 10.3389/fmed.2020.00100

**Published:** 2020-03-31

**Authors:** Arieh Gomolin, Elena Netchiporouk, Robert Gniadecki, Ivan V. Litvinov

**Affiliations:** ^1^Division of Dermatology, McGill University Health Centre, Montreal, QC, Canada; ^2^Division of Dermatology, University of Alberta, Edmonton, AB, Canada

**Keywords:** artificial intelligence, barriers, contact allergens, dermatology, melanoma, nevi, psoriasis, machine learning

## Abstract

Artificial intelligence (AI) has become a progressively prevalent Research Topic in medicine and is increasingly being applied to dermatology. There is a need to understand this technology's progress to help guide and shape the future for medical care providers and recipients. We reviewed the literature to evaluate the types of publications on the subject, the specific dermatological topics addressed by AI, and the most challenging barriers to its implementation. A substantial number of original articles and commentaries have been published to date and only few detailed reviews exist. Most AI applications focus on differentiating between benign and malignant skin lesions, however; others exist pertaining to ulcers, inflammatory skin diseases, allergen exposure, dermatopathology, and gene expression profiling. Applications commonly analyze and classify images, however, other tools such as risk assessment calculators are becoming increasingly available. Although many applications are technologically feasible, important implementation barriers have been identified including systematic biases, difficulty of standardization, interpretability, and acceptance by physicians and patients alike. This review provides insight into future research needs and possibilities. There is a strong need for clinical investigation in dermatology providing evidence of success overcoming the identified barriers. With these research goals in mind, an appropriate role for AI in dermatology may be achieved in not so distant future.

## Introduction

Dermatology is a field with the growing interplay of digitalization, telehealth, and informatics (1). The increasing presence of artificial intelligence (AI) worldwide, has led to numerous attempts to leverage this technology for dermatological applications (2). In a recent international survey of 1,271 dermatologists, 85.1% of responders were aware of AI as an emerging topic in their field yet only 23.8% had good or excellent knowledge on the subject (3). Moreover, 77.3% agreed that AI will improve dermatologic care and 79.8% thought that AI should be a part of medical training. Informing stakeholders on the current stance of AI is thus necessary to promote what dermatologists themselves believe to be a growing, beneficial and potentially obligatory aspect of the field. To date, many publications exist on specific AI topics in dermatology but few provide a basic overview and address the wide landscape. In this article, we summarize the status of the literature on AI in dermatology using three sections. First, we highlight the types of articles published on this subject. We then focus on dermatologic diseases targeted by AI, and finally, we spotlight the identified barriers impeding AI implementation.

## Types of Articles Published

### Original Research

The overwhelming majority of articles published to date are original research articles. These describe the design of AI applications that can perform dermatology-related tasks. For example, articles have studied tools that can segment a psoriasis lesion or differentiate between benign and malignant skin lesions (4, 5). Although these AI tools have not yet been implemented clinically, these papers describe their technological feasibility and identify the potential clinical relevance should they be further validated. Most of these studies are authored by engineering researchers with few dermatologists as co-authors. Although the number is increasing, relatively few papers involve significant dermatologist collaboration in conceiving, designing and interpreting the studies. To overcome known barriers to clinical implementation, partnership with dermatologists is key. Zakhem et al. highlight in their recent review of melanoma screening applications, that when dermatologists were involved in the study design, the AI applications leveraged significantly larger patient datasets that were more representative of true clinical scenarios (6). Another important form of collaboration is conducting prospective clinical trials and very few of these exist (7). One landmark study was conducted by Dreiseitl et al. (8). In their protocol, patients with undiagnosed pigmented lesions presented to a dermatology clinic and were assessed separately by both non-expert physicians using an AI device and by expert dermatologists. The study design therefore resembled a realistic clinical practice scenario. The results indicated inferiority of the automated system, and unfortunately, no similar studies have been published to date. Whether the lack of similar studies is a publication bias or a literature gap remains unclear.

### Reviews

A small number of systematic reviews exist at this time. Most of these cover the potential use of AI in differentiating between benign and malignant skin lesions. For example, studies have reviewed the specificities and sensitivities of AI tools for melanoma screening (9). To the best of our knowledge, only one systematic review has been published on dermatological applications of AI in general, not limited to neoplastic lesions (10).

### Commentaries

An extensive amount of commentaries exist on the topic of AI in dermatology. These papers either highlight the potential impact of AI or stress the challenges of its implementation (11, 12).

## Dermatological Applications of AI

### Keratinocyte Carcinomas and Melanoma

There is an abundant and growing body of research demonstrating the preliminary success of AI applications at distinguishing between benign nevi *vs*. melanoma (5, 13–34). The main principle behind these applications is that dermatoscopic or non-dermatoscopic images of lesions can be broken down into individual pixels for analysis. A representative example by Jafari et al. describes an application that examines images pixel by pixel and extracts 60 features from each to predict disease classification (24). These applications are typically validated by comparing their ability to correctly diagnose lesions to the ability of certified dermatologists (32). One review of photo recognition applications by Safran et al. included 48 melanoma-screening tools and demonstrated a mean sensitivity of 87.60% and a mean specificity of 83.54% (9). Interest toward this topic has grown to the extent that an international skin imaging competition was founded in 2016 and has been occurring annually since (32, 35). Although these applications have become more robust, prospective clinical trials are rare and known implementation barriers are continuously debated.

An increasing number of original studies have also begun classifying non-melanoma skin cancers (also known as keratinocyte carcinomas) vs. benign and pre-malignant lesions (36–44). For example, Spyridonos et al. developed an AI model that could differentiate between actinic keratosis and normal skin with a specificity of 89.8% and a sensitivity of 91.7% (37). Altogether, most of the research on the topic of skin cancer demonstrates technological feasibility combined with the growing evidence supporting clinical utility. What remains to be demonstrated is whether such tools can be implemented and relied upon in daily clinical practice.

AI has also been used beyond photo recognition. Rather than processing image pixels, applications can also process numerical values in various sequences and extract trends. For example, Tan et al. described an application that predicted the complexity of micrographic Mohs surgery based on variables assessed at the initial evaluation visit such as tumor size and patient age (43). They were able to create a preliminary model that could theoretically be used to triage patients and prioritize Mohs referrals. Although most of the research on AI and skin cancer is based on photo recognition algorithms, other opportunities exist.

### Ulcer Assessment

There is a growing body of research on diabetic and pressure ulcer applications (45–48). Thus, far most studies demonstrate methods for improving wound assessments using image recognition (45).

Articles have described applications capable of measuring precise wound boundaries, and differentiating between the types of tissue involved (45–47, 49). For example, Dhane et al. demonstrated an AI application's ability to segment the area of ill-defined ulcers with a sensitivity of 87.3% and specificity of 95.7% (47). Mukerjee et al. demonstrated an AI application's ability to classify granulation, slough and necrotic tissue with 87.61% accuracy (46). Risk prediction tools also exist. Alderden et al. described a tool that leverages data in the electronic health records of admitted patients, to predict their tendency to develop pressure ulcers (50). Altogether, these applications have preliminarily been shown to be technologically feasible, they have not yet been validated extensively in clinically trials.

### Psoriasis and Other Inflammatory Skin Diseases

Several original research articles exist on AI applications for inflammatory dermatoses. Most of these studies thus far have focused on improving psoriasis classification methods using image recognition (4, 51, 52). A representative example is a study conducted by Shrivasta et al., which compared the ability of several applications at classifying the severity of psoriasis lesions. The systems described achieved average sensitivities between 93.81 and 99.76% and average specificities between 97 and 99.99% (4).

Emam et al. described a psoriasis application beyond image recognition. They demonstrated a system's ability to predict psoriasis patient responses to biologic therapy using parameters gauged at an initial visit such as patient's weight and age of onset of psoriasis (53). They were able to create a preliminary model that could theoretically be used to optimize therapy for patients.

Beyond psoriasis, applications have been described classifying acne, lichen planus, pityriasis lichenoides and dermatomyositis (10, 54–58). Seite et al. developed a smartphone AI tool that grades and classifies types of acne lesions (e.g., comedonal, inflammatory, post inflammatory hyperpigmentation, etc.) (59). Huang et al. developed a multi disease classifier that could analyse 34 attributes (e.g., erythema, scaling, definite borders, etc.) and differentiate several papulosquamous diseases such as psoriasis, seborheic dermatitis, lichen planus, pityriasis, and chronic dermatitis (58).

Although most of these experiments relied on images of skin, one application assessed muscle ultrasound images and differentiated between normal muscle, dermatomyosisits, polymyositis, and inclusion body myositis with accuracies between 76.2 and 86.6% (60). Altogether, the theoretical utility of these applications for inflammatory diseases is significant, both further technological validation and clinical experimentation are needed.

### Predicting Skin Sensitization Substances

Research is also accumulating on using AI to minimize exposure to skin-sensitizing substances (61–65). A representative example by Zang et al. described an application capable of analyzing physiochemical properties of substances (e.g., melting point) and determining whether the substance could be a sensitizer or not (65). This application yielded an accuracy of 81% when the substances were studied in a human cohort. Wilm et al. reviewed current advances in skin sensitization testing and highlighted several other examples, where AI has provided a method to reduce animal testing (66). While this use of AI can have an impact on a population wide level, significant technological and clinical validation studies are necessary.

### Novel Applications in Pathology and Gene Expression Profiling

Applications have been described that can automate histology image processing and classification (67–71). For example, Arevalo et al. described a system that analyzes histopathological images and can classify basal cell carcinoma with 98.1% accuracy (67). Olsen et al. described a system that diagnosed dermal nevi and seborrheic keratosis with high accuracies and may serve as a future method to increase the efficiency of analyzing these prevalent benign tumors (72). Algorithms have also been described that can identify predictive genes and biomarkers for diseases (73–83). A representative example by Reimann et al., described an AI model capable of diagnosing psoriasis vulgaris based on the expression level of 4 genes with 96.4% accuracy (78). In another study assessing genetic differences in psoriasis genotypes, Patrick et al. used a combination of statistical learning and machine learning to identify new loci and predict the tendency of cutaneous psoriasis patients to develop psoriatic arthritis symptoms (84). While these investigations are in an early phase, the potential for their impact can be significant.

## Identified Barriers

### Choice of Predictive Model

AI algorithms are continually being developed and each has advantages and challenges. Beam et al. discussed the relationship of AI compared to more classic statistical models (85). They detail how predictive technologies can be viewed on a machine-learning spectrum. Statistical models are lower on the spectrum because humans impose assumptions and guide many aspects of the algorithm. True machine learning is highest on the spectrum because the algorithms evolve without human involvement. A systematic review by Christodoulou et al. found no objective advantage of machine learning compared to longitudinal regression for binary clinical prediction (86). However, the review does summarize various theoretical reasons why machine learning may be superior to longitudinal regression in certain instances such as processing data with a strong signal to noise ratio (e.g., handwriting) or with a significant number of predictor subcategories (e.g., images) (86). There are therefore many unanswered questions regarding whether advanced forms of AI are actually needed or if more primitive technologies can accomplish the same tasks.

### Generalizability

One of the main limitations to AI is that the decisions made by these technologies are ultimately a reflection of the input data used to train the system (87). This theoretically implies that applications can only be used reliably in populations they were trained to assess. If applications are trained in one population and tested in another, the results are technically not generalizable and are subject to systematic biases such as overfitting. For example, Han et al. experimented with a skin cancer detection algorithm and concluded that overall performance could be improved if trained with a wide variety of data from multiple ethnic populations (36). However, simply using more data does not necessarily solve this problem. For example, Navarrete-Dechent et al. took Han et al.'s established an AI algorithm that was trained with a relatively diverse set of data and tested it in a unique database of Caucasian Americans from the southern United States. They found that the performance was suboptimal compared to how it was reported originally (88). The issue of generalizability is thus not simple to solve and may require either unique or extended data depending on the composition of the population being tested. This tendency for systematic bias has numerous implications for dermatology given the various demographic factors that affect making a diagnosis such as age, gender, race, and ethnicity to name a few.

### Standardization

Even if the application is trained using data from the correct population, images of new lesions need to be comparable. What angle should the image be taken at? What lighting should the room have? What should the background be? Are there pen markings? These are factors that can affect decision-making by AI. For example, a study by Winkler et al. showed that surgical markings significantly interfered with the ability of a system to correctly diagnose dermatoscopic images of melanoma and increased the false positive rate (89). Artificial intelligence relies on standardization and there are numerous non-standardized aspects to dermatology unlike in other specialties (e.g., radiology) (90). Although databases are intentionally large to account for variability, factors such as these create an infinite possibility for divergence.

### Data Requirements

One large barrier is the prerequisite for copious quantities of data of appropriate quality to power AI algorithms (91). A growing effort in the United States has been to solve this barrier using DataDerm, the American Academy of Dermatology's electronic health record system. Worldwide collaboration is likely required to achieve the ideal scenario where all of the necessary data categories are represented.

### Interpretability

Artificial intelligence algorithms are formed, re-evaluated and constantly changing without human input. This is why the technology has often been termed a “black box” technology (92). Although AI is therefore flexible and can theoretically accomplish more than humans and human-guided statistical algorithms, many aspects and certainly the logic behind the decision-making is often not interpretable. When a certified dermatologist conducts a personalized assessment and arrives at a conclusion, that conclusion can be rationalized and explained based on existing clinical evidence. At this time, decisions made by AI cannot be interpreted in this way. This is a strong limitation which influences whether society and regulatory bodies will accept it in the daily practice of medicine.

### Acceptance

A proper history followed by a physical examination in a well-lit examining room, while assessing for texture and eliciting specific signs for a given lesion (e.g., Darier, dimple, buttonhole signs, etc.) complemented by additional investigations/imaging or a biopsy is a standard way to establish a diagnosis in dermatology. Furthermore, it is accepted that while some diagnoses are clinical, others rest solely on histologic findings or a combination of clinical and histologic results correlation. This holistic approach cannot be fully replaced by computer programs and this is felt to be one of the most important barriers to implementing AI (93). Many patients also want to see and partner with a physician who is vested in helping them and may not be satisfied with isolated computerized tools (12).

### Liability

There is also the issue of liability (94). If AI is relied upon and an adverse outcome ensues, is the dermatologist responsible? With this in mind, a common belief is that AI will only become a guidance tool and not an absolute diagnostic tool.

### Next Steps

To address these barriers, several broad recommendations have been made to date. One clear need is for prospective clinical trials. If the question is whether AI can improve a dermatologic clinical encounter then studies that revolve around the clinical encounter are crucial (7). Dermatologist collaboration has also been highlighted as essential (6). Systems need to be trained with the full spectrum of human populations and clinical presentations that challenge dermatologists in clinical practice (88). Systems can also benefit from receiving inputs on other metrics available to physicians such as anatomic location, duration of the lesion and images of unaffected skin (88). Standardization practices also need to be implemented for photographing new lesions. Finally, given the lack of interpretability of many AI applications, we hypothesize that improving the lay descriptions of the algorithms and study designs can lead to improved acceptance by physicians and society at large. This would also aid regulatory decision makers who will need to adopt stances on liability.

## Conclusion

AI is being increasingly studied in dermatology. Although most applications involve analyzing and classifying images, there are other tools such as risk assessment calculators. The most progress thus far has taken place in the field of melanoma diagnosis, followed by ulcer and psoriasis assessment tools, then followed by numerous less frequently studied applications. However, critical barriers and literature gaps exist that significantly limit AI's applicability to clinical practice at this time. For the less common applications, technological papers and commentaries are needed to improve capabilities and provoke interest. For the more saturated topics, there is a larger need for clinical trials providing evidence of clinical efficacy, while successfully overcoming the identified barriers. With these research goals in mind, an appropriate role for AI in dermatology may be achieved.

## Author Contributions

AG, EN, RG, and IL performed the literature review and wrote the paper. RG and IL supervised the project.

### Conflict of Interest

The authors declare that the research was conducted in the absence of any commercial or financial relationships that could be construed as a potential conflict of interest.
